# NEIGHBORHOOD CHARACTERISTICS INFLUENCE OLDER ADULTS' HEALTH AND MENTAL HEALTH OUTCOMES

**DOI:** 10.1093/geroni/igac059.1091

**Published:** 2022-12-20

**Authors:** Weidi Qin, Weidi Qin

## Abstract

Both social and physical aspects of neighborhood characteristics are related to a wide range of health and mental health outcomes. There has been increasing evidence pointing to the link between neighborhood-level factors and health among older adults. Specifically, older adults living in disadvantaged neighborhoods with under-resourced infrastructure may experience more daily activity limitations, mental health symptoms, and increased morbidity and mortality. Positive aspects of the neighborhood, such as social cohesion, may serve as a social capital resource and protect against adverse health outcomes. On the contrary, negative aspects of the neighborhood, such as physical disorder, can be a substantial stressor leading to poor health. The neighborhood environment also disproportionally affects racial and ethnic minorities in the US. This symposium session will present four studies exploring important topics related to neighborhood factors of health among older adults. Collectively, the findings will inform neighborhood-level interventions to promote health and well-being among community-dwelling older adults. This session will start with a talk by Dr. Chan on the link between neighborhood and disability across six ethnic groups of older Asian Americans residing in New York City. Dr. Perry will present a qualitative study to explore the environmental and infrastructure challenges in the neighborhood from the perspective of older adults in Detroit. This will be followed by Dr. Jiang’s talk on the relationship between neighborhood cohesion and mortality among a sample of older Chinese in Chicago. Finally, the session will conclude with Dr. Qin’s presentation on how neighborhood characteristics affect older adults’ mental health trajectories.

